# Single-Cell RNA Sequencing of Tocilizumab-Treated Peripheral Blood Mononuclear Cells as an *in vitro* Model of Inflammation

**DOI:** 10.3389/fgene.2020.610682

**Published:** 2021-01-05

**Authors:** Arya Zarinsefat, George Hartoularos, Dmitry Rychkov, Priyanka Rashmi, Sindhu Chandran, Flavio Vincenti, Chun J. Yee, Minnie M. Sarwal

**Affiliations:** ^1^Department of Surgery, University of California, San Francisco, San Francisco, CA, United States; ^2^Department of Bioengineering and Therapeutic Sciences, University of California, San Francisco, San Francisco, CA, United States; ^3^Department of Medicine, University of California, San Francisco, San Francisco, CA, United States

**Keywords:** single cell RNA seq, COVID-19, tocilizumab (IL-6 inhibitor), kidney transplantation, transcriptomics, bioinformatics and computational biology

## Abstract

COVID-19 has posed a significant threat to global health. Early data has revealed that IL-6, a key regulatory cytokine, plays an important role in the cytokine storm of COVID-19. Multiple trials are therefore looking at the effects of Tocilizumab, an IL-6 receptor antibody that inhibits IL-6 activity, on treatment of COVID-19, with promising findings. As part of a clinical trial looking at the effects of Tocilizumab treatment on kidney transplant recipients with subclinical rejection, we performed single-cell RNA sequencing of comparing stimulated PBMCs before and after Tocilizumab treatment. We leveraged this data to create an *in vitro* cytokine storm model, to better understand the effects of Tocilizumab in the presence of inflammation. Tocilizumab-treated cells had reduced expression of inflammatory-mediated genes and biologic pathways, particularly amongst monocytes. These results support the hypothesis that Tocilizumab may hinder the cytokine storm of COVID-19, through a demonstration of biologic impact at the single-cell level.

## Introduction

Coronavirus disease 2019 (COVID-19), caused by the severe acute respiratory syndrome coronavirus 2 (SARS-CoV-2), has posed a significant threat to global health since emerging at the end of 2019. Although the spectrum of symptomatic infection ranges significantly, and most infections are not severe (Chan et al., 2020; Huang et al., 2020; Wang et al., 2020), the overall global burden of the disease has been significant with up to nearly 20% mortality in certain geographic/demographic groups (Onder et al., 2020; Wu and McGoogan, 2020). While notable progress has been made in the understanding the virology and disease process, the abrupt onset and lack of effective vaccination has made treatment of COVID-19 difficult (Ahmed et al., 2020; Mitja and Clotet, 2020).

Interleukin (IL)-6 is a key regulatory cytokine for the innate and adaptive immune response and is a growth factor for B cell proliferation and differentiation, an inducer of antibody production, and a regulator of CD4 + T cell differentiation (Hunter and Jones, 2015; Jordan et al., 2017). Early data from the COVID-19 outbreak has shown that the complications from the disease are partly due to increases in various cytokines, including IL-6 (Chen et al., 2020; Mehta et al., 2020; Vaninov, 2020; Yang et al., 2020), and that elevated IL-6 levels may be associated with worse outcomes (Chen et al., 2020; Li et al., 2020; Wan et al., 2020). Tocilizumab is an IL-6 receptor antibody, which binds to both the membrane-bound and soluble forms of the IL-6 receptor (IL-6R), thereby inhibiting the action of the cytokine/receptor complex and interfering with the cytokine’s effects (Lee et al., 2014). It is a well-studied and accepted therapy for rheumatoid arthritis (Campbell et al., 2011; Singh et al., 2011; Smolen and Aletaha, 2011), and has also been studied in giant cell arteritis (Kwan and Thyparampil, 2020) and organ transplantation (Choi et al., 2017; Jordan et al., 2017; Shin et al., 2020). As such, multiple global investigators are currently undertaking clinical trials to further assess the efficacy of Tocilizumab in the treatment of COVID-19 and its complications (ClinicalTrials.gov). Thus far, it has been shown that COVID-19 patient plasma inhibits the expression of HLA-DR which may be partially restored by Tocilizumab treatment, and that treatment with Tocilizumab may also improve lymphopenia associated with COVID-19 (Giamarellos-Bourboulis et al., 2020). Preliminary data for Tocilizumab treatment on COVID-19 outcomes has shown improvement in clinical outcomes (Luo et al., 2020; Xu et al., 2020). While the clinical effects of Tocilizumab in inflammatory and autoimmune disease has been well-studied, there is a paucity of data on the mechanistic/biologic impact of the drug on our immune system.

Given the current state of the COVID-19 epidemic and possible efficacy of IL-6/IL-6R inhibition with the use of Tocilizumab, we believed a deeper analysis of the mechanistic/biologic effects of Tocilizumab could further elucidate the effects of the drug on our immune system. Herein we present an analysis of the impact of Tocilizumab on immune cells using single-cell RNA sequencing (scRNA-seq). We map the response of peripheral blood mononuclear cell (PBMC) subsets to cellular activation using CD3/CD28 stimulation (Luo et al., 2019; Miragaia et al., 2019; Pizzolato et al., 2019; Szabo et al., 2019; Cai et al., 2020). Relevant to understanding the impact of Tocilizumab in suppressing immune activation and inflammation, as seen in the COVID-19 response, we examined the effect of Tocilizumab on stimulated cells, as part of an investigator-initiated clinical trial in kidney transplant (KT) recipients with subclinical rejection (*NIAID U01 AI113362-01*^1^). Given that our samples are from transplant recipients with subclinical graft rejection, we believed that by utilizing PBMCs from these patients, we would be looking at cells from an environment that at baseline has an increased inflammatory burden. By further stimulating these cells, we hoped to best recreate an *in vitro* model to represent the presence of a cytokine storm. We provide a resource characterizing the effect of Tocilizumab on immune cells at a single-cell level, and demonstrate the unique and unexpected impact of Tocilizumab on monocytes, and how its effect on suppressing inflammation may be further augmented based on the resting versus activated state of PBMCs before exposing the cells to IL-6R inhibition.

## Materials and Methods

### Sample Collection

This study was performed as part of an ancillary to a randomized controlled clinical trial of 15 KT recipients that were diagnosed with subclinical rejection on their 6-month post-transplant protocol biopsy and randomized to either continue standard of care (Tacrolimus, mycophenolate, and steroid) immunosuppression (control arm, 8 patients) or standard of care plus Tocilizumab (Tocilizumab treatment arm, 7 patients). There were 10 male and 5 female patients included in the study, with a roughly equal proportion of males/females within each arm of the study (5 of 8 patients in the control arm were males, and 5 of 7 patients in the Tocilizumab arm were males). Patients in the treatment arm were given Tocilizumab at a dose of 8 mg/kg IV every 4 weeks, for a total of 6 doses. Patients in both arms of the study had blood collected at baseline prior to the initiation of Tocilizumab (in the treatment arm patients), then at 3, 6, and 12 months after the start of the study, for a total of 4 blood samples per all 15 patients in the study. PBMCs were isolated from blood samples by Ficoll-Paque^TM^ PLUS density gradient centrifugation (GE Healthcare, Chicago, IL, United States) and frozen in fetal bovine serum (Gibco, Waltham, MA, United States) containing 10% (vol/vol) dimethyl sulfoxide (Sigma-Aldrich, St. Louis, MS, United States). Cells were frozen and not thawed until the day of the experiment when they were used directly for *in vitro* stimulation.

### Stimulation With Anti-CD3 and Anti-CD28 Antibodies

Frozen PBMCs were thawed, four vials at a time to ensure maximum cell recovery, in a water bath at 37 Celsius. Cells were counted using a hemocytometer, split in half, and were then adjusted to 2 × 10^5^ cells/well and triplicate plated in multiscreen 96-well plates (Falcon, Corning, NY). Cells were stimulated with soluble anti-CD3 (5 μg/mL; MabTech, Cincinnati, OH, United States) and anti-CD28 antibodies (10 μg/mL; MabTech, Cincinnati, OH, United States) at 37 Celsius, 5% CO_2_ for 24 h. Unstimulated PBMCs were incubated under identical conditions to reduce any confounding from incubation conditions other than stimulation. Since all PBMCs were split in half prior to any downstream processing, all samples from control and Tocilizumab-treated patients at all study time points were both stimulated and not stimulated as part of the study design.

### Sample Processing

After overnight stimulation/incubation, the cells were harvested and counted using a hemocytometer and orange acridine solution. Any cell suspension that was less than 25 cells/μL was disqualified from multiplexing due to low cell counts. A total of 90 samples were collected over the 2 days of experiments with 4 samples being disqualified due to low cell counts. Multiplexing cell pools were designed such that no pair of stimulated and unstimulated samples from the same patient were in the same pool and such that no samples from the same collection time point were in the same pool. The same number of cells from each patient and experimental condition were multiplexed into their respective pools to make a final total of 300,000 cells per pool. Any remaining non-pooled cells were resuspended in RNAlater (Thermo-Fisher, West Sacramento, CA, United States) and saved for SNP array. Cell pools were then centrifuged at 400 *g* for 5 min and media was aspirated. Cell pellet was resuspended in a small volume of Wash Buffer (0.4% BSA in 1XPBS) and the suspension was filtered through a 40 μM cell strainer (Falcon, Corning, NY, United States).

### Library Construction and Sequencing

scRNA-seq libraries were prepared using the 10× Chromium Single Cell 3′ Reagent Kits v3, according to the manufacturer’s instructions. Briefly, the isolated cells were washed once with PBS + 0.04% BSA and resuspended in PBS + 0.04% BSA to a final cell concentration of 1000 cells/μL as determined by hemocytometer. Cells were captured in droplets at a targeted cell recovery of 4000–8000 cells, resulting in estimated multiplet rates of 0.4–5.4%. Following reverse transcription and cell barcoding in droplets, emulsions were broken and cDNA purified using Dynabeads MyOne SILANE (Thermo-Fisher, West Sacramento, CA, United States) followed by PCR amplification (98°C for 3 min; 12–16 cycles of 98°C for 15 s, 67°C for 20 s, 72°C for 1 min; 72°C for 1 min). Amplified cDNA was then used for 3′ gene expression library construction. For gene expression library construction, 2.4–50 ng of amplified cDNA was fragmented and end-repaired, double-sided size selected with SPRIselect beads (Beckman Coulter, West Sacramento, CA, United States), PCR amplified with sample indexing primers (98°C for 45 s; 14–16 cycles of 98°C for 20 s, 54°C for 30 s, 72°C for 20 s; 72°C for 1 min), and double-sided size selected with SPRIselect beads. Pooled cells were loaded in a 10× chip in three replicate wells such that each well contained 50,000 cells. Given the large number of cells and large number of patient samples, the entire experiment and sequencing was performed in 2 separate batches to prevent cell death during counting. Each day resulted in 4 unique pools with each pool run in triplicate wells for sequencing. Sequencing single-cell RNA libraries were sequenced on an Illumina NovaSeq S2 to a minimum sequencing depth of 50,000 reads/cell using the read lengths 26 bp Read1, 8 bp i7 Index, 91 bp Read2.

### Demultiplexing

To assign cells to donors of origin in our multiplexed design, we used the genetic demultiplexing tools *freemuxlet* and sample matching script, each being part of the *popsicle* suite of population genetics tools^2^. *Freemuxlet* leverages the genetic polymorphisms present in transcripts and clusters the droplet barcodes to assign each to a given donor (or assign them as doublets between donors). The algorithm returns these droplets with donor assignments and a set of variants per donor. These sets of variants are then matched using genotypic similarity to those from an external genotyping SNP array to determine which patient is which donor.

Due to memory constraints, the *freemuxlet* algorithm was run in 3 batches, divided by the experimental day and pool of patients processed. We show the distribution of singlets across the batches (Supplementary Figure 1A; bar plots, top) and the genotypic similarity between *freemuxlet*-annotated donors and patients (Supplementary Figure 1A; heatmaps, bottom). Upon initially examining the data, we noted two inconsistencies between the data and experimental design: (Chan et al., 2020) two patients (patients 5 and 6, both healthy control patients) had identical genotypes, and (Huang et al., 2020) patient 2 had very low cell numbers. The first inconsistency is almost certainly due to human error during the sample submission or running of the genotyping array, since these patients were not identical twins nor related in any way. The second inconsistency is likely due to low cell viability or inaccurate cell counting or pooling of patient 2′s cells. To rectify the first inconsistency, we recognized the absence of patient 5 in the design of experimental day 2 pools. Because patient 5 was not included in the pools and patient 6 was, and because the patient 5/6 genotype was still detected, we concluded that that genotype assayed in the array is actually patient 6′s. Given this, through process of elimination, we were able to assign donor 10′s cells in the Day 1 data to patient 5. To rectify the second inconsistency, we opted to input one less donor into the *freemuxlet* algorithm, such that it would not attempt to cluster patient 2′s cells and would instead identify them as ambiguous. We show that these remediations do not change the expected linear relationship between doublet rate and total cell-containing droplets (Supplementary Figure 1B). Apart from those inconsistencies, there was a 1-to-1 mapping of donors to patients, and through those remediations we were able to definitively assign a detected genotype to all detected individuals. After assignment of each droplet barcode to patients, droplet barcodes were then filtered to remove doublet droplets containing cells from multiple individuals, and the remaining singlets were analyzed as described below.

### Data Analysis

Raw FASTQ files were processed using *CellRanger* (v 3.0.1) to map reads against human genome 38 as a reference, filter out unexpressed genes, and count barcodes and unique molecular identifiers (UMIs). Subsequent analyses were conducted with *Seurat* (v 3.1.2) (Butler et al., 2018) in *R* (v 3.6.2). We compared stimulated control cells, to stimulated Tocilizumab-treated cells from 3 to 6 months post-treatment with Tocilizumab. Utilizing *Seurat*, we first filtered cells to only keep those that had less than 10% mitochondrial genes and cells with numbers of features greater than 200 and less than 2,500. Cells were assigned patient identification based on the *freemuxlet* output described above, and once patients were identified, additional treatment/stimulation/time metadata could be applied. Given that our experiment was divided over 2 days given the high number of samples/cells, we applied *Seurat’s* SCTransform function for data integration to account for any possible batch effects from experiment days (Hafemeister and Satija, 2019; Stuart et al., 2019). Once the data was integrated, we continued downstream data processing. We first determined the principal components (PCA), then constructed a shared nearest neighbor graph (SNN), identified clusters with a resolution of 0.75, and finally visualized the cells using uniform manifold approximate and projection (UMAP), per the typical Seurat workflow (Butler et al., 2018). Clustering was achieved by using 15 components from the PCA dimensionality reduction.

To identify cluster-specific markers following the creation of UMAP plots, we utilized normalized RNA counts of all clusters, scaled the data, and performed differential gene expression (DE) testing by applying the Wilcoxon rank sum test using *Seurat’s* FindMarkers function (Butler et al., 2018). We also plotted normalized and scaled gene expression of canonical markers in conjunction with DE testing to determine identities of each cluster. To compare control vs. Tocilizumab-treated cell clusters from specific cell types (such as monocytes, CD4 + T cells, or CD8 + T cells), we once again utilized normalized/scaled RNA counts and performed DE testing with FindMarkers.

To perform pathway analysis (PA) for any specific comparison we performed, we filtered for all differentially expressed genes with an adjusted (based on the Bonferroni correction) *p*-value < 0.05, and then selected the top 10 percentile of genes with the highest log-fold changes. These top genes were used to perform the PA utilizing the Reactome database (Fabregat et al., 2017) with the *clusterProfiler* package (Yu et al., 2012). We also performed analyses of enriched biological processes utilizing the Gene Ontology database for these same groups of cells (Balakrishnan et al., 2013). To perform cell trajectory analysis, we first subset our clusters and cell types of interest from our *Seurat* workflow, then performed dimensionality reduction and cell ordering with *Monocle* (Qiu et al., 2017) (v 2.14.0). We were then able to plot specific cells by their trajectory branches based on their pseudotime values assigned by *Monocle*. DE of individual cell trajectory branches was then performed with *Monocle’s* BEAM (branched expression analysis modeling) function, followed by visualization of these differentially expressed branches with *Monocle’s* heatmap visualization tool.

We then leveraged two publicly available bulk RNA-seq datasets from PBMCs, GSE152418 (Arunachalam et al., 2020), and peripheral blood monocytes, GSE160351 (Brunetta et al., 2020), of COVID-19 patients and healthy individuals. The raw gene counts of the GSE152418 dataset were downloaded and normalized by the variance stabilizing transformation approach using the *R* package *DESeq2* (Love et al., 2014), and the pre-normalized gene counts of GSE160351 dataset were downloaded. The Ensembl gene IDs were converted to gene symbols using the *R* package *biomaRt* (Durinck et al., 2009). We then filtered these datasets to only include our upregulated control monocyte genes that we obtained using the *FindMarkers* function as described above. Once we had our gene list of upregulated monocyte genes, we performed unsupervised hierarchical clustering of the above COVID-19 datasets, using the *pheatmap* package (Kolde, 2012).

## Results

**FIGURE 1:**
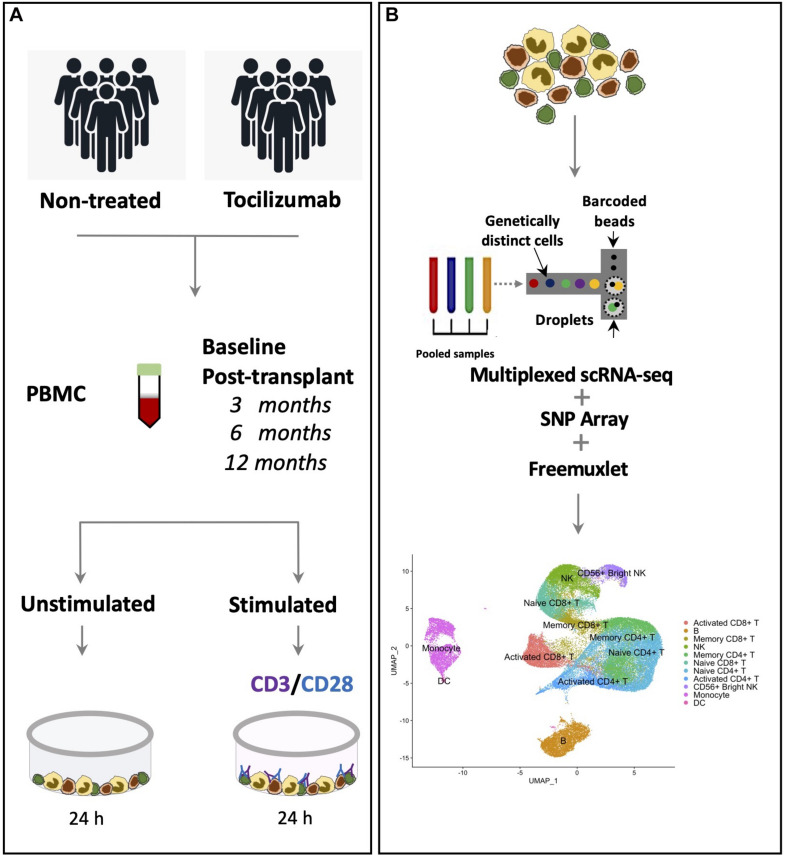
Experimental design layout. **(A)** Experimental design showing treatment groups, collection of PBMCs at study baseline, then 3, 6, and 12 months post-treatment, and *in vitro* stimulation of PBMCs with CD3/CD28 stimulating antibodies. **(B)** Multiplexed droplet scRNA-seq and SNP array of PBMCs, with application of *freemuxlet* tools for demultiplexing and patient assignment, with eventual downstream cell clustering and annotation.

**FIGURE 2:**
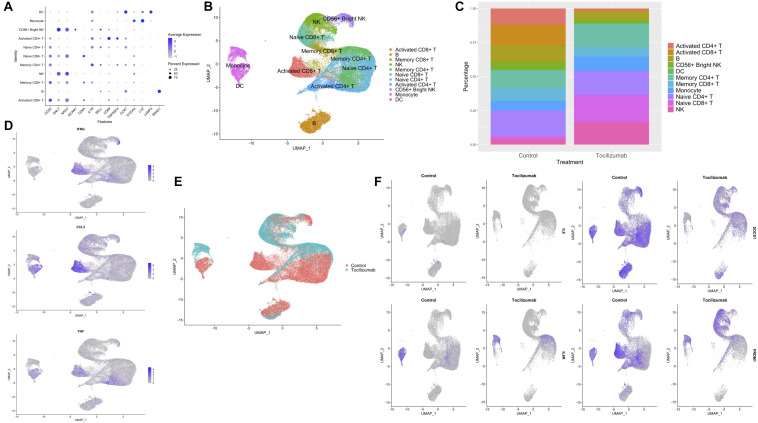
UMAP projections, cell subset annotation, and expression of inflammatory markers and IL6R pathway genes in control vs. Tocilizumab-treated PBMCs. **(A)** Dot plot of canonical markers used for annotation of the 20 cell clusters. Average feature expression represented by color gradient with lower expression represented by light gray, and higher expression represented by blue. Size of dots represent the percent of cells within that specific cluster that express the feature of interest. **(B)** UMAP with final cell type annotations. **(C)** Bar plot showing the percentage of each cell type in control vs. Tocilizumab-treated groups. **(D)** Feature plots showing expression of select cytokines involved in SARS-CoV-2 cytokine storm (*IFNG*, *CCL3*, and *TNF*) based on control vs. Tocilizumab treatment status. Feature expression represented by color gradient, with high expression represented by blue and low expression represented by gray. **(E)** UMAP with cell clusters identified based on control vs. Tocilizumab treatment status. **(F)** Feature plots showing expression of *IL6*, *IL6R*, and downstream IL6R pathway genes (*SOCS1, PRDM1*) based on control vs. Tocilizumab treatment status. Feature expression represented by color gradient, with high expression represented by blue and low expression represented by gray.

The overall experimental design is presented in graphical form (Figure 1). In order to examine the impact of Tocilizumab on the composition and expression of circulating single cells, we compared scRNA-seq data from stimulated control cells (patients not treated with Tocilizumab) PBMCs, to stimulated cells after 3 to 6 months of Tocilizumab treatment. After filtering cells, a total of 57,737 cells remained for analysis. These cells were put through our analysis pipeline described (see section “Materials and Methods”). After UMAP projection of cell clusters, there were a total of 21 distinct clusters representing major PBMC groups. Cluster 20 was found to express canonical markers from multiple PBMC cell types, signifying this was likely a cluster of doublets that had not removed by our previously performed cell filtering. Clusters were then annotated according to canonical cell type markers (Figure 2A), which are also demonstrated as feature plots to show the relative expression amongst the different clusters (Supplementary Figure 2). Cluster 2 expressed markers of CD8 + T cells, and additionally markers of memory T cell expansion (Patil et al., 2018), while clusters 6 and 15 lacked memory cell markers and were therefore identified as naïve CD8 + T cells. Clusters 4 and 5 expressed markers of both CD4 + T cells and memory T cell expansion [*S100A4, IL7R* (Salek-Ardakani and Croft, 2006; Martin and Badovinac, 2018)]. Clusters 0, 8, and 16 expressed markers of CD4 + T cell activation [*TNFRS4*, *CD69* (Simms and Ellis, 1996)]. Clusters 3 and 17 lacked *CD3D* expression, but expressed *GNLY* (Tewary et al., 2010), suggesting they were NK cell clusters, while cluster 10 additionally expressed *CD56*, suggesting this was a CD56 + bright NK cell cluster (Michel et al., 2016). Clusters 11 and 13 expressed *CD14*, *CD16*, and *LYZ*, signifying these were monocyte clusters (Mukherjee et al., 2015; Sampath et al., 2018). Cluster 18 expressed *LAMP3* and was therefore identified as a DC cluster (Yin et al., 2017). Clusters 1 and 19 expressed *MS4A1* and were therefore identified as B cells (Zuccolo et al., 2013). This assignment of cell types resulted in our final annotated clusters (Figure 2B).

After 6 months of treatment with Tocilizumab there is a shift in peripheral blood subset frequencies observed across no treatment (control) vs. treatment (Tocilizumab) groups. In comparison to changes in overall cell types, there was little observed effect on frequencies of naïve CD4 + /CD8 + T cells, DC, or NK cells, but with a marked reduction of activated CD4 + T cells (approximately 12.5% of control PBMCs were activated CD4 + T cells, while there were essentially no activated CD4 + T cells in the Tocilizumab group, Figure 2C). Feature plots showing the expression of “cytokine storm” (Tisoncik et al., 2012) related pro-inflammatory genes are cell-type specific, with predominance for expression in T cell and monocyte clusters (Figure 2D). Although many genes are known to be involved in the cytokine storm of COVID-19 (Chua et al., 2020; Ye et al., 2020), we demonstrate that some of the key pro-inflammatory genes (cytokines, interferons, and tumor necrosis factor) are also noted as part of the inflammatory profile in control (no Tocilizumab) patients (Figure 2D, control cells). Overall, stimulated PBMCs not exposed to Tocilizumab show T cell activation signals. Within these different cell subsets, Tocilizumab therapy results in significant polarization of gene expression based on UMAP presentation (Figure 2E), with notable polarization by treatment status observed in monocytes. Because our analysis focused on an *in vitro* cytokine storm that was represented by CD3/CD28 stimulation, we did not focus our analysis on unstimulated cells. Of note, when we did look at unstimulated Tocilizumab-treated vs. control cells, we did not observe the same notable polarization or differential expression of genes seen between different cell types in the stimulated Tocilizumab-treated vs. control cells. We also looked for sex-based differences in cell clustering, and did not find any notable differences based on sex (Supplementary Figure 3).

Given Tocilizumab’s function as an IL-6R blocker, we looked at the expression of *IL6*, *IL6R*, as well as *SOCS1* [feedback inhibitor of IL-6 signaling, expressed upon IL-6 pathway activation (Prêle et al., 2008)], and *PRDM1* [activated by the *JAK/STAT3* pathway via activation of the IL-6 pathway (Garbers et al., 2015; Liu et al., 2019)] in Tocilizumab-treated cells (Figure 2F). Tocilizumab-treatment resulted in the expected reduction of *IL6R*, *SOCS1*, and *PRDM1* expression, in CD4 + and CD8 + T cells, and unexpectedly also in monocytes. *IL6* expression did not appear to be affected by Tocilizumab treatment.

**FIGURE 3:**
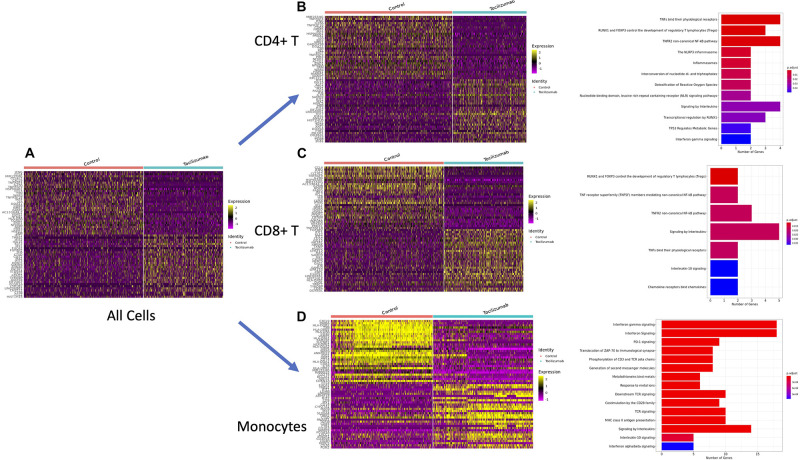
Differential expression testing and pathway analysis of all cells, CD4 + T cells, CD8 + T cells, and monocytes. **(A)** Heatmap of top 30 genes with highest log-fold changes in all control and Tocilizumab-treated cells. **(B)** Heatmap of top 30 genes with highest log-fold changes in all CD4 + T control and Tocilizumab-treated cells, with corresponding PA of top 10% most highly differentially expressed genes (based on log_2_-fold change) in control vs. Tocilizumab cells. **(C)** Heatmap of top 30 genes with highest log-fold changes in all CD8 + T control and Tocilizumab-treated cells, with corresponding PA of top 10% most highly differentially expressed genes (based on log_2_-fold change) in control vs. Tocilizumab cells. **(D)** Heatmap of top 30 genes with highest log-fold changes in all control and Tocilizumab-treated monocytes, with corresponding PA of top 10% most highly differentially expressed genes (based on log_2_-fold change) in control vs. Tocilizumab cells. Gene expression level represented by color gradient ranging from purple (low expression) to yellow (high expression). PA figure x-axis represents the number of genes from each pathway that was present in the gene list. Adjusted *p*-values for pathway enrichment are represented as a color gradient with larger *p*-values colored blue and smaller *p*-values colored red.

We then looked at the top 30 most differentially expressed genes (highest log_2_-fold changes) for control vs. Tocilizumab cells to create heatmaps of gene expression amongst all cells (Figure 3A), CD4 + T cells (Figure 3B), CD8 + T cells (Figure 3C), monocytes (Figure 3D). We then took the top tenth percentile of genes with the highest log_2_-fold changes and performed corresponding PA for these genes utilizing the Reactome database. PA showed enrichment of inflammatory pathways such IL and TNF signaling amongst control cells. Looking at the most differentially expressed genes (highest log_2_-fold changes) for control vs. Tocilizumab monocytes (Figure 3D), we saw some notable differences as would be expected. Control monocytes were enriched in chemokines such as *CXCL9*, various HLA genes involved in antigen processing (Yamamoto et al., 2020) (*HLA-DQB1*, *HLA-DRB5*), *CD40* [member of the TNF-receptor superfamily (Martínez et al., 2020)], and *SOCS1* [downstream gene activated by IL-6R pathway, as previously discussed (Prêle et al., 2008)]. PA revealed enrichment of many inflammation-related pathways, including interferon, interleukin, T cell receptor (TCR), and PD-1 signaling in control PBMCs, suggesting the relative suppression of these pathways in cells exposed to Tocilizumab (Figure 3A). In CD4 + and CD8 + T cells, we also found enrichment of inflammatory pathways (Figures 3B,C), such as inflammasomes and interleukin signaling, although the overall number of enriched pathways was fewer than seen amongst monocytes. Enriched pathways for these cell types are also shown in table form (Supplementary Figure 4). Additionally, we looked at enriched biological process pathways in CD4 + T cells, CD8 + T cells, and monocytes. We found that across all cell subsets, there was a propensity for enrichment of inflammation-related processes in control vs. Tocilizumab-treated cells (Supplementary Figure 5).

**FIGURE 4:**
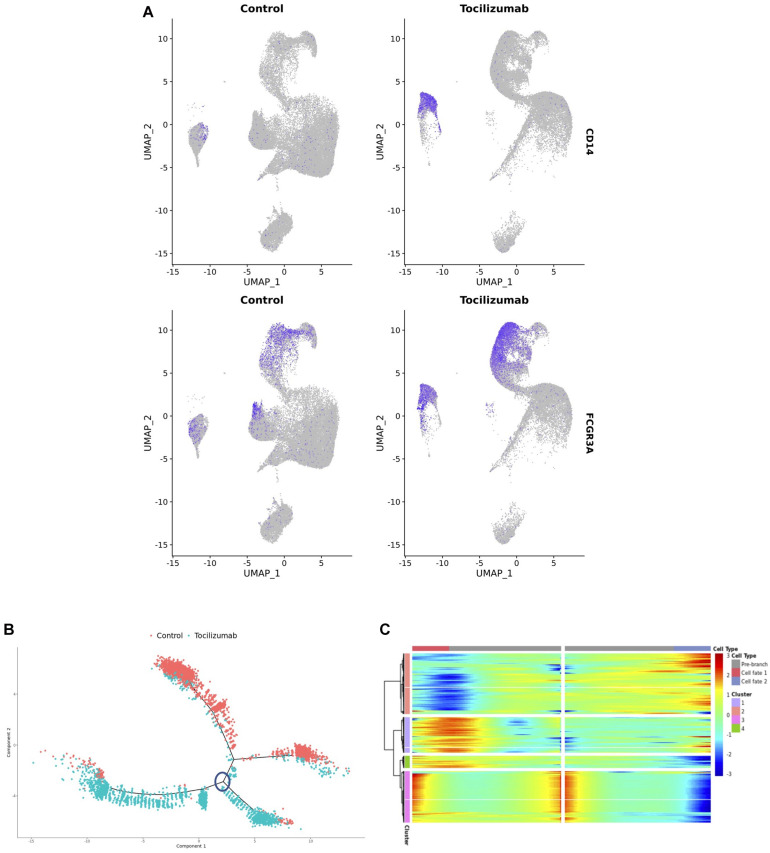
Differential expression testing, cell trajectory analysis, and pathway analysis of monocyte subsets. **(A)** Feature plots showing expression of CD14 and CD16 based on control vs. Tocilizumab treatment status. Feature expression represented by color gradient, with high expression represented by blue and low expression represented by gray, with higher CD14 expression noted in Tocilizumab cells. **(B)** Cell trajectory analysis of monocyte clusters showing distinct lineages of control vs. Tocilizumab cells; blue circle represents branch point used in subsequent heatmap analysis. **(C)** Heatmap from branched expression analysis modeling for most differentially expressed genes between branch points from c (analyzed branch point marked by blue circle), showing clusters of differentially expressed genes between branches. Gene expression represented as color gradient from blue (low expression) to red (high expression). Cell type annotation represented by two separate cell fates as seen in **(B)** with middle of heatmap representing the start of pseudotime and clear separation of control vs. Tocilizumab cell fates.

In addition to the effect of Tocilizumab on T cells, we also observed an unexpected polarization of monocytes after Tocilizumab treatment (Figure 2E). Notably, the Tocilizumab monocyte cluster was enriched for *CD14*, suggestive of an increased presence of classical monocytes (Mukherjee et al., 2015), while *CD16*/*FCGR3A* expression was more evenly expressed between the two clusters (Figure 4A). The 2,000 most highly variable features amongst the monocytes in our dataset were then utilized to perform a cell trajectory analysis. These monocyte features were input into the *Monocle* pipeline to create cell trajectories, and annotated based on treatment status. This revealed six distinct cell trajectory branches, with two of the branches containing nearly all control cells, and the other four branches containing nearly all Tocilizumab-exposed PBMCs (Figure 4B). As *Monocle* tracks changes as a function of progress along the trajectory, the distinct branches containing nearly all control cells vs. Tocilizumab-treated cells, supports the idea that there are unique transcriptional changes amongst the cells after patient exposure to IL6-R blockade. We utilized *Monocle’s* BEAM function to perform branched expression analysis modeling of the distinct cell trajectory branches for Tocilizumab-exposed PBMCs (circled branch, Figure 4B), which showed distinct clusters of cells based on treatment status (Figure 4C).

Finally, we mapped the upregulated control cell monocyte genes to COVID-19 bulk RNA-seq gene expression data from PBMCs (Arunachalam et al., 2020) and monocytes (Brunetta et al., 2020) and visualized results with heatmaps created using unsupervised hierarchical clustering. We found that our upregulated monocyte gene list, when applied to COVID-19 patients, showed nearly perfect clustering when applied to the PBMC dataset (Supplementary Figure 6A), and perfect clustering when applied to the monocyte dataset (Supplementary Figure 6B), with regards to whether or not patients had COVID-19, or were healthy.

## Discussion

The results of this study showed that in PBMCs undergoing a cytokine storm signal in rejection (Sarwal et al., 2003), with overlapping signatures of *IFNG*, *CCL3*, and *TNF* expression, along with TCR signaling also seen in the cytokine storm of COVID-19 (Chua et al., 2020; Ye et al., 2020), there is suppression of these inflammatory pathways after Tocilizumab treatment. This is inclusive of suppression of downstream signaling of IL6-R pathway genes in both monocytes and T cells. Our study was focused on the simulation of an *in vitro* cytokine storm model by CD3/CD28 stimulation of PBMCs that were either Tocilizumab-treated or control cells. While our findings here describe our findings from stimulated cells, it is worth noting that we did not observe any notable polarization of cells, or significant differential gene expression of identical cell types based on treatment status, when looking just at unstimulated cells. This was suggestive that it was under stimulated conditions where the effects of Tocilizumab treatment on PBMCs were most notable.

Monocytes have been shown to play a significant role in the pathophysiology of COVID-19 (Merad and Martin, 2020).

A significant expansion of populations of monocytes producing IL-6 has been observed in the peripheral blood of patients with COVID-19 in ICUs compared with those patients who did not require ICU hospitalization (Zhou et al., 2020), with similar findings of increased IL-6 production from monocytes also seen by scRNA-seq analysis of PBMCs (Wen et al., 2020). With regards to Tocilizumab treatment and COVID-19, multiple centers have found that Tocilizumab treatment has been associated with improved outcomes, and that measured IL-6 tended to decrease after Tocilizumab treatment in patients with improved outcomes, while IL-6 tended to increase in those with worse outcomes (Luo et al., 2020; Xu et al., 2020). This suggests that Tocilizumab may in fact counteract the cytokine storm seen in COVID-19, by decreasing activity of IL-6. Guo et al. performed a single-cell analysis of two patients with severe COVID-19 pre and post-treatment with Tocilizumab, looking at differences in gene and pathway enrichment amongst monocytes (Guo et al., 2020). Interestingly, the authors found enrichment of genes related to regulation of the acute inflammatory response, regulation of leukocyte activation, cell chemotaxis, and the cellular response to chemokines in severe-stage COVID-19 patients compared to remission-stage patients and healthy controls, suggesting that the inflammatory storm caused by monocytes is suppressed by Tocilizumab treatment. Our findings were similar to Guo et al. in that we have an enrichment of similar inflammation-mediated pathways amongst control cells that had not received Tocilizumab. Our findings are from the first clinical trial utilizing Tocilizumab for transplant rejection recipients and the first scRNA-seq analysis for such a study. We show a separation of cell clustering based on treatment status, reduced enrichment of inflammatory pathways in Tocilizumab patients, and relatively reduced expression of IL-6R pathway genes in Tocilizumab-treated cells. As would be expected, we did not observe any differences in IL-6 gene expression between control and Tocilizumab cells (as Tocilizumab is an IL-6R blocker), but rather only effects on the subsequent function of that cytokine’s pathways. We also show an enrichment of *CD14* expression (associated with classical monocytes) in Tocilizumab-treated monocytes, which are believed to be phagocytic, but with reduced inflammatory attributes (Mukherjee et al., 2015). This is consistent with our PA described above that shows enrichment of inflammatory pathways in control cells, but not Tocilizumab-treated cells (possibly due to the increased presence of non-inflammatory classical monocytes in Tocilizumab-treated cells).

Interestingly, when we utilized our upregulated genes from control cells in monocytes as the gene list for performing unsupervised hierarchical clustering of gene expression data from COVID-19 PBMCs and monocytes, we saw both perfect and near perfect clustering based on patient phenotype. This suggests that the inflammatory pathway genes that are upregulated in stimulated control cells from our study, may in fact be representative of some of the same genes that are affected by COVID-19 infection.

Our study is limited by the lack of COVID-19 patients and the *in vitro* nature of our inflammation model. Our goal was to better understand the biologic effects of Tocilizumab and its impact on inflammation, and while our group does not have single-cell data for Tocilizumab treatment in COVID-19 patients, we demonstrate the anti-inflammatory effects of Tocilizumab. This data shows promise that in this *in vitro* model, Tocilizumab does have anti-inflammatory effects which may be of clinical and biologic interest in actual COVID-19 patients. Despite these limitations, we believe that transferability of our findings exist. Specifically, the finding of the gene list of upregulated monocyte genes from control cells which leads to perfect clustering of COVID-19 vs. healthy patients, is suggestive of a common pathway of inflammatory genes. In addition, this was seen in both COVID-infected PBMCs and monocytes, suggesting that even across different cell types, there may be a set of common upregulated genes in inflammation in COVID. Interestingly, single-cell analyses of COVID monocytes has shown inflammatory gene signatures such as increased expression of *IFNG*, similar to what we saw in our control cells (Schulte-Schrepping et al., 2020). Our future work may include single-cell analysis of COVID-19 transplant patients that have received Tocilizumab for treatment, which would help us to better understand these biologic mechanisms in actual COVID-19 patients.

Our findings, in conjunction with the available data on clinical outcomes of Tocilizumab treatment (Xu et al., 2020) and ongoing trials, show promise for the use of Tocilizumab in the treatment of patients with COVID-19. The results of our study support the belief that Tocilizumab may be effective in reducing the inflammatory burden that results in the adverse outcomes of COVID-19. Future studies will need to be undertaken to look at outcomes of Tocilizumab treatment for COVID-19 in a clinical trial setting, ideally in conjunction with scRNA-seq analysis of these patient’s blood samples to achieve a greater understanding of the transcriptomic effects of infection and treatment at a single-cell level.

## Data Availability Statement

The data presented in the study are deposited in the GEO repository, accession number GSE163014 and https://www.ncbi.nlm.nih.gov/geo/query/acc.cgi?acc=GSE163014.

## Ethics Statement

The studies involving human participants were reviewed and approved by UCSF IRB. The patients/participants provided their written informed consent to participate in this study.

## Author Contributions

AZ was responsible for data cleaning, analysis, interpretation, and manuscript writing. GH was responsible for data cleaning, analysis, and methodology. DR was responsible for data analysis and interpretation. PR was responsible for experimental design and performance of experiments. SC was responsible for clinical data and clinical trial design. FV was responsible for clinical data and clinical trial design. CY was responsible for study design, review, oversight of analysis, and manuscript edits. MS was responsible for study design, review, oversight of analysis, and manuscript edits. All authors contributed to the article and approved the submitted version.

## Conflict of Interest

The authors declare that the research was conducted in the absence of any commercial or financial relationships that could be construed as a potential conflict of interest.

## Footnotes

1 https://grantome.com/grant/NIH/U01-AI113362-06

2 https://github.com/statgen/popscle

## Supplementary Material

Supplementary Figure 1 *Freemuxlet* patient assignment and doublet rates. **(A)** Bar plots showing the distribution of singlets across the batches of samples, with the *x*-axis representing the *freemuxlet-*assigned patient ID and the *y*-axis representing singlet counts (top panel); heatmaps representing the genotypic similarities between *freemuxlet*-annotated donors and patients, with the *x*-axis representing patient IDs based on SNP array and the *y*-axis representing their freemuxlet ID (bottom panel). **(B)** Scatter plot showing the expected linear increase in doublet rate with the increase in singlets; *x*-axis represents the total number of singlets and doublets and the *y*-axis represents the corresponding doublet rate.

Supplementary Figure 2 Feature plots of canonical markers used for cluster annotation. Genes expression represented by color gradient, with highest expression represented by blue, and lowest expression represented by gray.

Supplementary Figure 3 UMAP of all cells, annotated by patient sex. UMAP plot of all cells, annotated by patient sex; although there is a greater proportion of male cells based on trial including more males, there is no clear pattern of clustering based on patient sex.

Supplementary Figure 4 Reactome pathway analysis tables. **(A)** Monocyte Reactome pathway analysis table with enriched Reactome pathways. **(B)** CD4 + T cell Reactome pathway analysis table with enriched Reactome pathways. **(C)** CD8 + T cell Reactome pathway analysis table with enriched Reactome pathways; columns included are Reactome pathway name, number of enriched genes that are present for that respective pathway, gene ratio (the number of genes present in the respective pathway divided by the total number of genes in the pathway), and the adjusted *p*-value for significant pathways; only pathways with an adjusted *p*-value < 0.05 were included.

Supplementary Figure 5 Gene ontology biological processes enrichment. **(A)** Enriched biological processes based on the Gene Ontology database for CD4 + T cells. **(B)** Enriched biological processes based on the Gene Ontology database for CD8 + T cells. **(C)** Enriched biological processes based on the Gene Ontology database for monocytes. Legend shows color gradient for adjusted *p*-values, with red being smaller adjusted *p*-values and blue being larger adjusted *p*-values. The *x*-axis represents the number of genes from the gene list that were a part of that respective biological process pathway.

Supplementary Figure 6 Heatmaps of COVID-19 and healthy patient gene expression. **(A)** Unsupervised hierarchical clustering of gene expression data from bulk RNA-seq of COVID-19 infected and healthy patient PBMCs, using only upregulated genes from control monocytes from our single-cell study; near perfect clustering is seen between COVID-19 and healthy patients; additional phenotypes included are patient sex and disease severity. **(B)** Unsupervised hierarchical clustering of gene expression data from bulk RNA-seq of COVID-19 infected and healthy patient monocytes, using only upregulated genes from control monocytes from our single-cell study; perfect clustering is seen between COVID-19 and healthy patients; relative gene expression is represented as a color gradient with higher gene expression represented in red and lower gene expression represented as blue.
