# Supplementary figures and images for: Single-Cell RNA Sequencing of Tocilizumab-Treated Peripheral Blood Mononuclear Cells as an in vitro Model of Inflammation
Source: Front Genet. 2021 Jan 5;11:610682. doi: 10.3389/fgene.2020.610682 (PMC7813999; doi:10.3389/fgene.2020.610682)

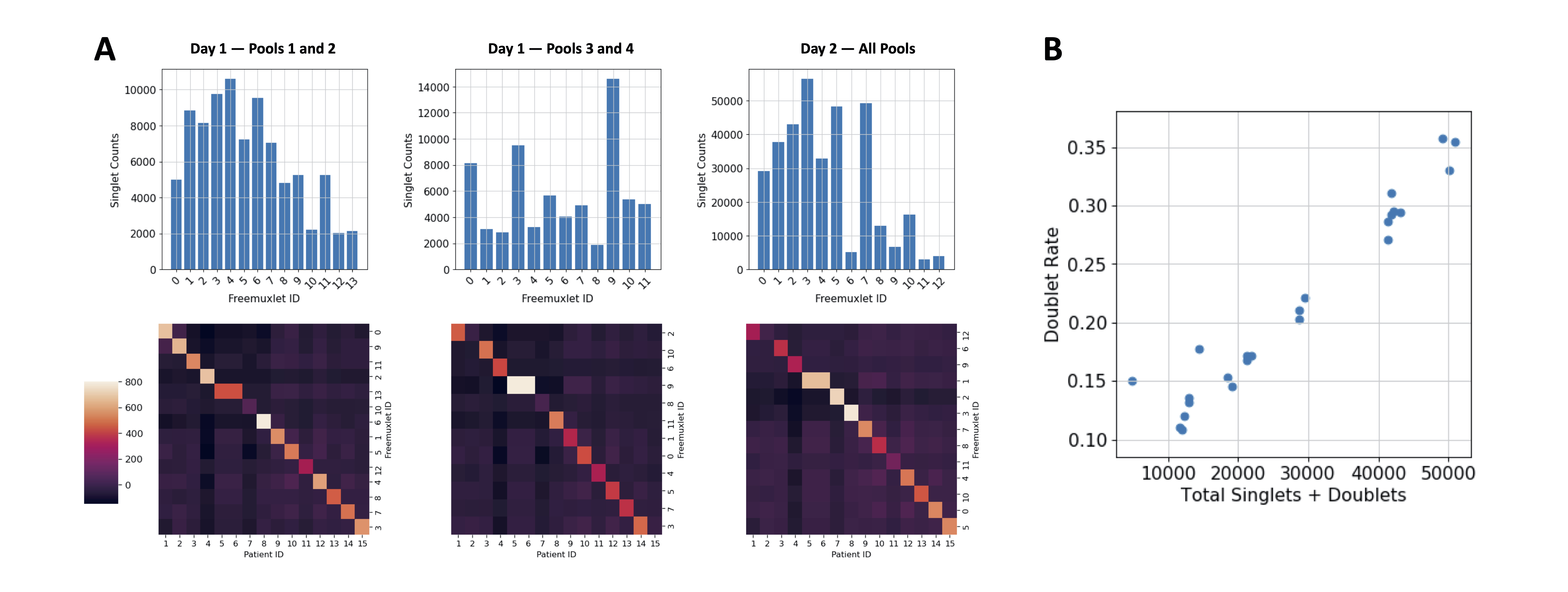

Supplement: Supplementary Figure 1 — Freemuxlet patient assignment and doublet rates. (A) Bar plots showing the distribution of singlets across the batches of samples, with the x-axis representing the freemuxlet-assigned patient ID and the y-axis representing singlet counts (top panel); heatmaps representing the genotypic similarities between freemuxlet-annotated donors and patients, with the x-axis representing patient IDs based on SNP array and the y-axis representing their freemuxlet ID (bottom panel). (B) Scatter plot showing the expected linear increase in doublet rate with the increase in singlets; x-axis represents the total number of singlets and doublets and the y-axis represents the corresponding doublet rate. [file Image_1.JPEG]

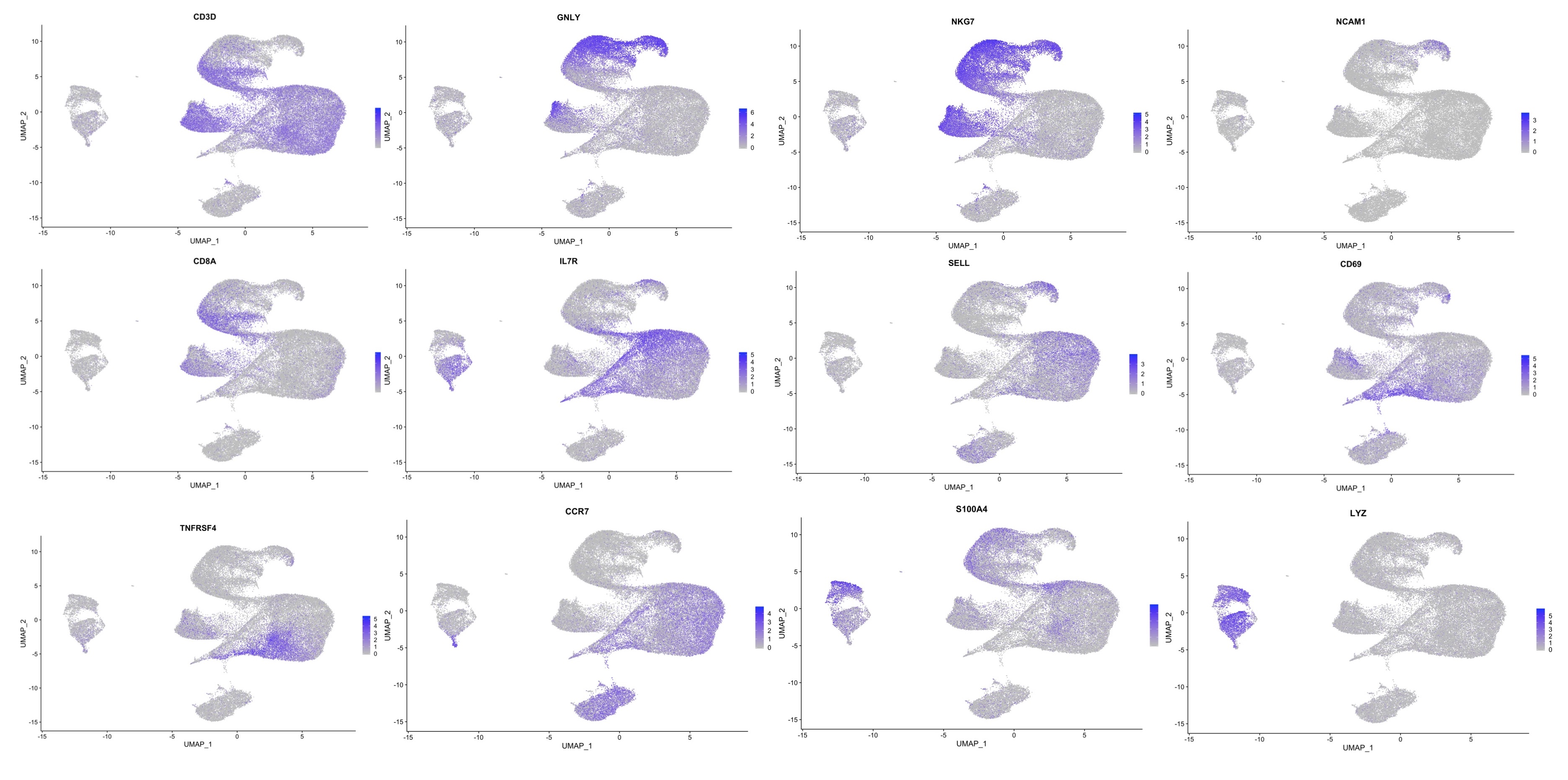

Supplement: Supplementary Figure 2 — Feature plots of canonical markers used for cluster annotation. Genes expression represented by color gradient, with highest expression represented by blue, and lowest expression represented by gray. [file Image_2.JPEG]

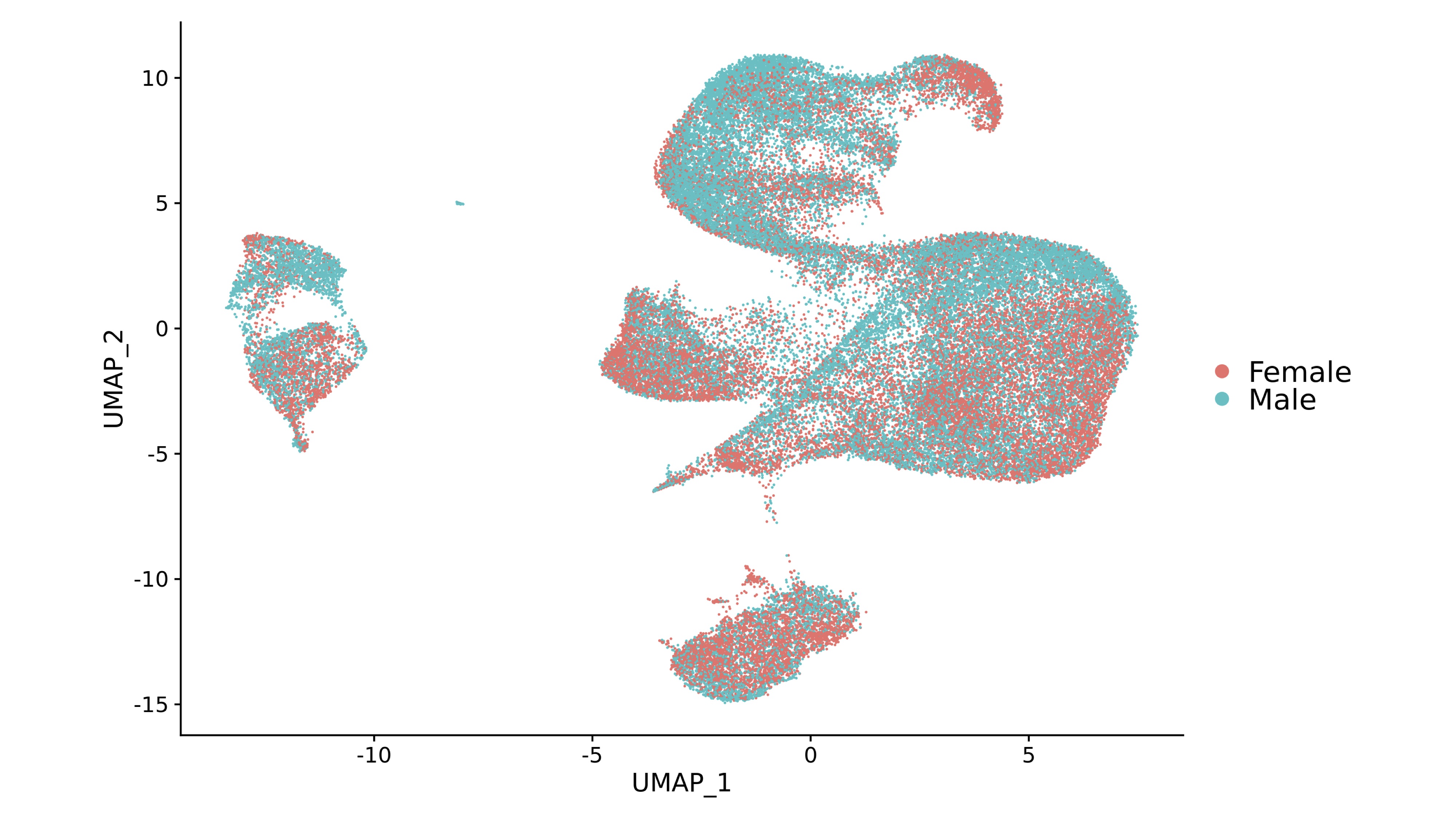

Supplement: Supplementary Figure 3 — UMAP of all cells, annotated by patient sex. UMAP plot of all cells, annotated by patient sex; although there is a greater proportion of male cells based on trial including more males, there is no clear pattern of clustering based on patient sex. [file Image_3.JPEG]

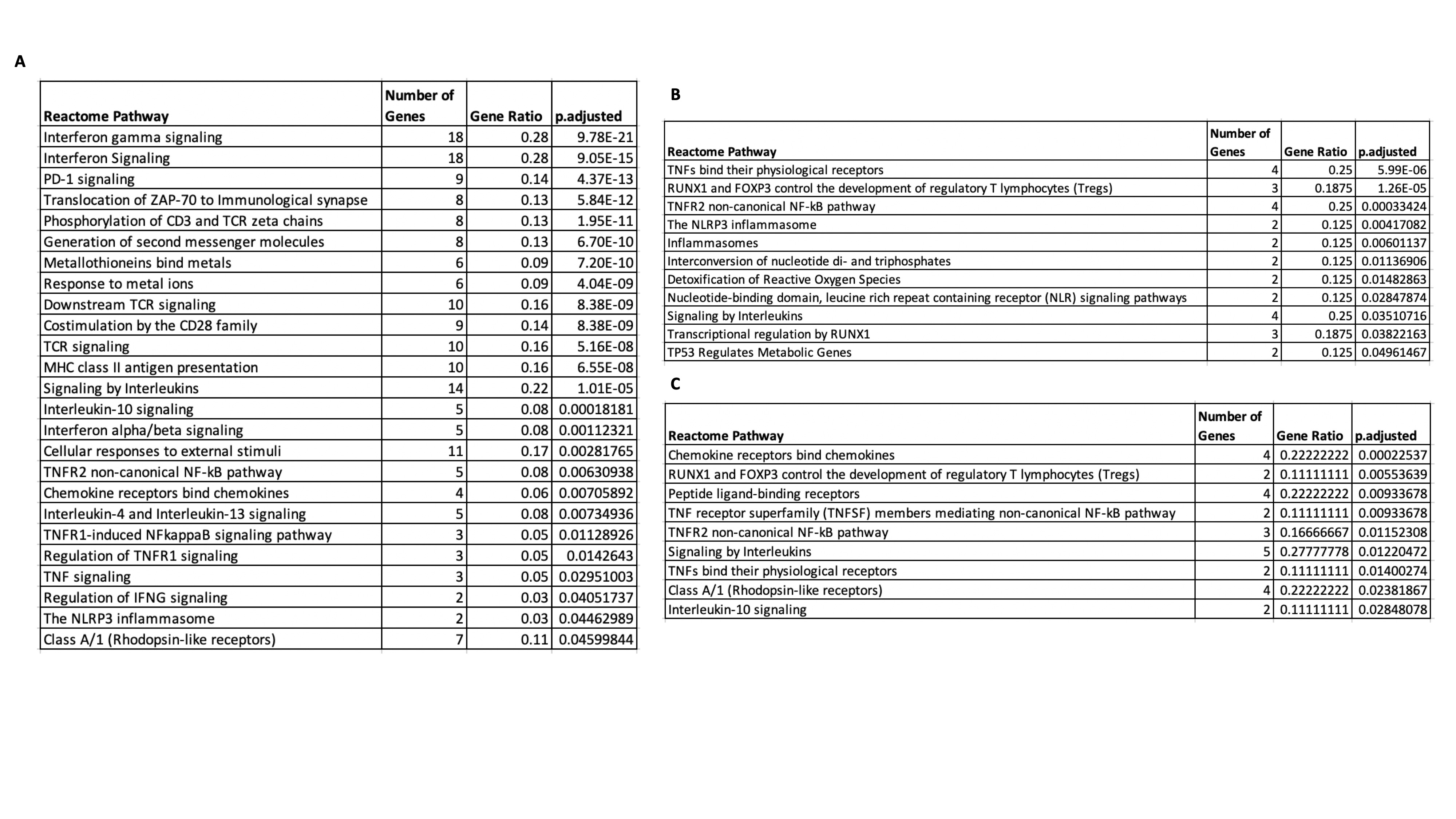

Supplement: Supplementary Figure 4 — Reactome pathway analysis tables. (A) Monocyte Reactome pathway analysis table with enriched Reactome pathways. (B) CD4 + T cell Reactome pathway analysis table with enriched Reactome pathways. (C) CD8 + T cell Reactome pathway analysis table with enriched Reactome pathways; columns included are Reactome pathway name, number of enriched genes that are present for that respective pathway, gene ratio (the number of genes present in the respective pathway divided by the total number of genes in the pathway), and the adjusted p-value for significant pathways; only pathways with an adjusted p-value < 0.05 were included. [file Image_4.JPEG]

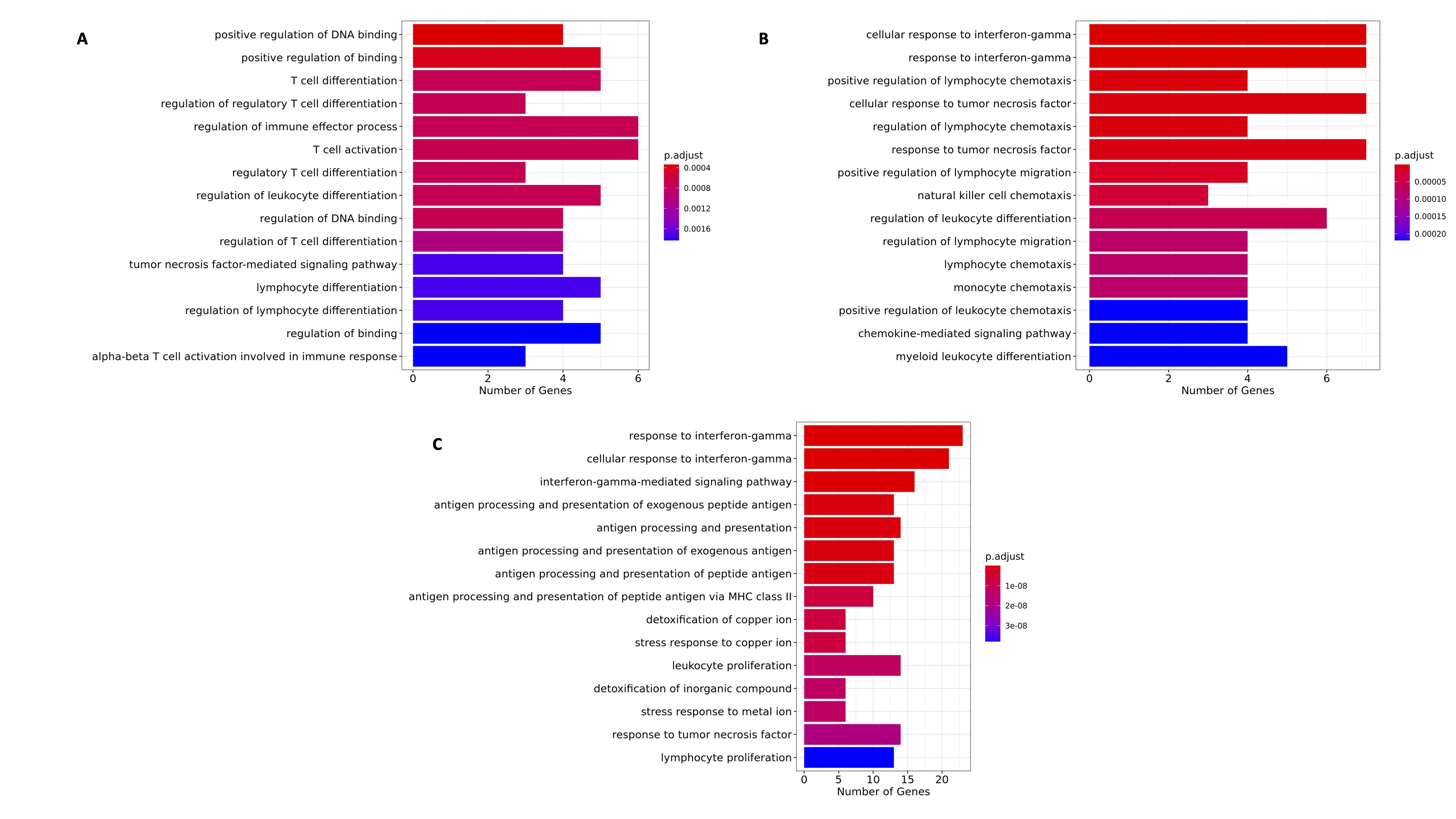

Supplement: Supplementary Figure 5 — Gene ontology biological processes enrichment. (A) Enriched biological processes based on the Gene Ontology database for CD4 + T cells. (B) Enriched biological processes based on the Gene Ontology database for CD8 + T cells. (C) Enriched biological processes based on the Gene Ontology database for monocytes. Legend shows color gradient for adjusted p-values, with red being smaller adjusted p-values and blue being larger adjusted p-values. The x-axis represents the number of genes from the gene list that were a part of that respective biological process pathway. [file Image_5.JPEG]

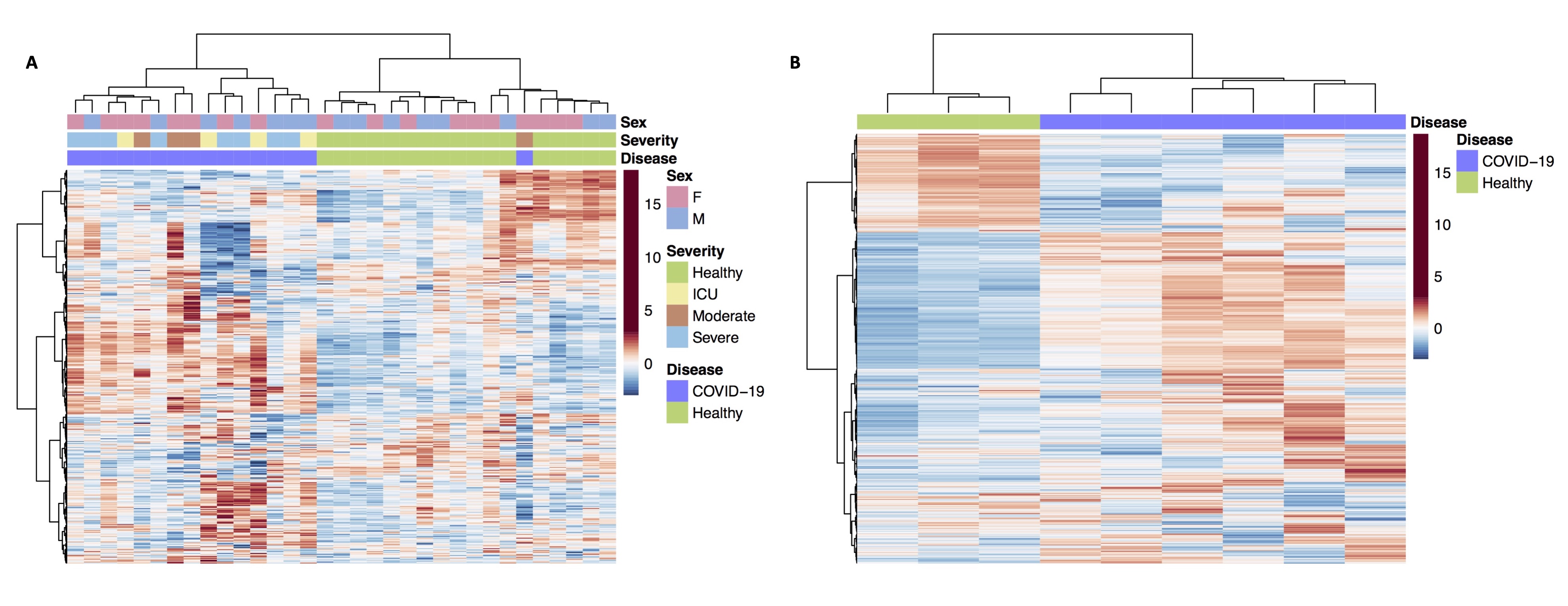

Supplement: Supplementary Figure 6 — Heatmaps of COVID-19 and healthy patient gene expression. (A) Unsupervised hierarchical clustering of gene expression data from bulk RNA-seq of COVID-19 infected and healthy patient PBMCs, using only upregulated genes from control monocytes from our single-cell study; near perfect clustering is seen between COVID-19 and healthy patients; additional phenotypes included are patient sex and disease severity. (B) Unsupervised hierarchical clustering of gene expression data from bulk RNA-seq of COVID-19 infected and healthy patient monocytes, using only upregulated genes from control monocytes from our single-cell study; perfect clustering is seen between COVID-19 and healthy patients; relative gene expression is represented as a color gradient with higher gene expression represented in red and lower gene expression represented as blue. [file Image_6.JPEG]
